# An Epileptic Seizure Prediction Method Based on CBAM-3D CNN-LSTM Model

**DOI:** 10.1109/JTEHM.2023.3290036

**Published:** 2023-06-27

**Authors:** Xiang Lu, Anhao Wen, Lei Sun, Hao Wang, Yinjing Guo, Yande Ren

**Affiliations:** College of Electronic and Information EngineeringShandong University of Science and Technology 74789 Qingdao Shandong 266590 China; Taian Second Hospital of Traditional Chinese Medicine Qingdao Shandong 271000 China; The Affiliated Hospital of Qingdao University Qingdao Shandong 266003 China

**Keywords:** CBAM, EEG, LSTM, seizure prediction, 3DCNN

## Abstract

Epilepsy as a common disease of the nervous system, with high incidence, sudden and recurrent characteristics. Therefore, timely prediction of seizures and intervention treatment can significantly reduce the accidental injury of patients and protect the life and health of patients. Epilepsy seizures is the result of temporal and spatial evolution, Existing deep learning methods often ignore its spatial features, in order to make better use of the temporal and spatial characteristics of epileptic EEG signals. We propose a CBAM-3D CNN-LSTM model to predict epilepsy seizures. First, we apply short-time Fourier transform(STFT) to preprocess EEG signals. Secondly, the 3D CNN model was used to extract the features of preictal stage and interictal stage from the preprocessed signals. Thirdly, Bi-LSTM is connected to 3D CNN for classification. Finally CBAM is introduced into the model. Different attention is given to the data channel and space to extract key information, so that the model can accurately extract interictal and pre-ictal features. Our proposed approach achieved an accuracy of 97.95%, a sensitivity of 98.40%, and a false alarm rate of 0.017 h^−1^ on 11 patients from the public CHB-MIT scalp EEG dataset. ***Clinical and Translational Impact Statement***—Timely prediction of epileptic seizures and intervention treatment can significantly reduce the accidental injury of patients and protect the life and health of patients.

## I. Introduction

Epilepsy is a chronic neurological disease in which sudden abnormal discharges of brain neurons lead to transient dysfunction of the brain. Because of the aberrant discharge’s various starting positions and modes of transmission. Epilepsy can present as paroxysmal movement, sensory, autonomic nerve, awareness, and mental abnormalities, among other complicated and varied clinical symptoms. At present, epilepsy has become the second largest neurological disease after headache. According to statistics, there are about 70 million patients with epilepsy worldwide, it increases by about 2 million people per year. Although epilepsy patients after regular antiepileptic drugs, surgery, nerve stimulation treatment, about 70 % of patients with seizures can be controlled, but there are still about 30 % of patients with intractable epilepsy do not have the appropriate treatment. For these patients, Epilepsy not only causes great burden to their lives and psychology, but also may endanger their life safety. Therefore, it is of great significance to predict the onset of epilepsy and to treat patients with drugs or nerve stimulation in advance to prevent them from harm.

**FIGURE 1.**
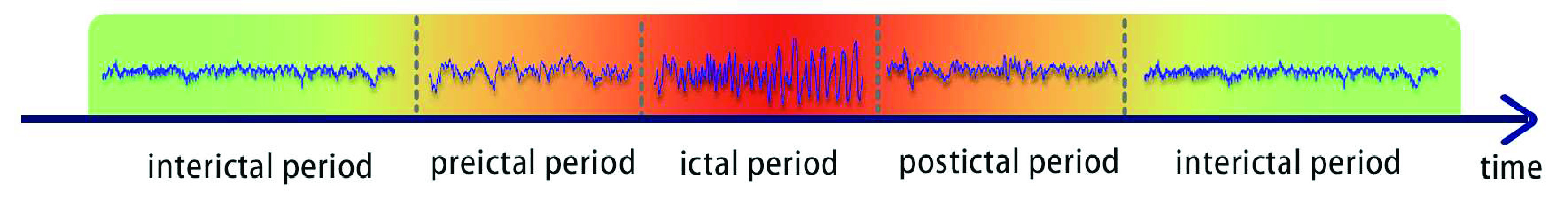
Four states of EEG in patients with epilepsy.

EEG is a graph created by boosting and capturing the brain’s natural biological potential from the scalp using sophisticated electronic equipment. It is the rhythmic and uninhibited electrical activity of networks of brain cells that has been captured by electrodes. EEG testing is particularly accurate for the diagnosis of epilepsy because it may precisely record scattered slow waves, spikes, or erratic spikes during seizures. EEG can be roughly divided into two types according to the acquisition mode: One is scalp EEG, and the other is intracranial EEG. According to EEG, doctors generally divide the EEG signals of epileptic patients into four periods, as shown in Fig. 1. Epileptic seizures are the time from the beginning to the end of seizures, usually lasting a few seconds or minutes. The preictal period is a few minutes to dozens of minutes before the onset of seizures. The postictal period is a period of time from the end of seizures to the return to normal in patients with epilepsy. The interictal period is a period of time between the late onset and the next pre-seizure, at this time the patient ’ s state is no different from normal. Studies have shown that the onset of epilepsy is sudden, but it is not an instantaneous attack, but from the normal state to the onset of a transition time, that is preictal period. Therefore, accurate identification of pre-seizure is of great significance for epilepsy prediction.

**FIGURE 2.**
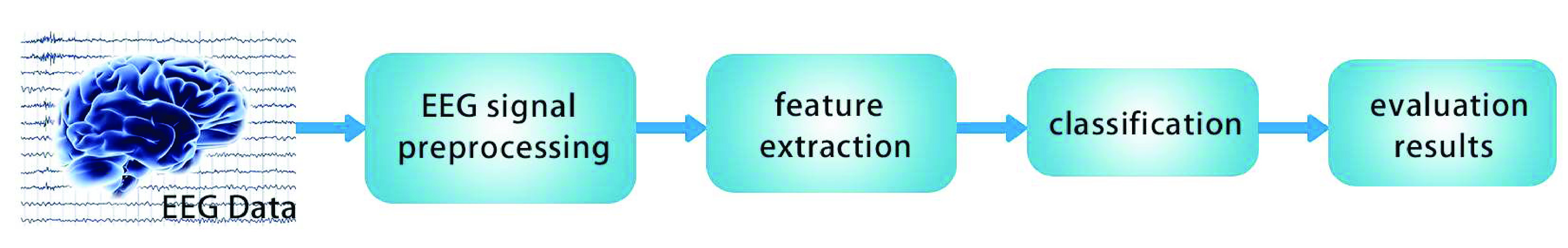
Flow chart of seizure prediction.

According to Fig. 2, the seizure prediction process normally entails data collection, EEG signal preprocessing, feature extraction, classification, and evaluation of the results. Epileptic seizure prediction is classified by different characteristics of EEG signals in preictal period and interictal periods. Seizure prediction dates back to the early 1870s, Viglione et al. first used patient ’s EEG to predict epilepsy [1]. In the 1980 s, Rogowski et al. [2] and Salant et al. [3] proposed an autoregressive model to analyze the parameter change information generated within 6 seconds before the onset, and introduced the physical-mathematical theory of nonlinear systems. This is a new method for predicting seizures. In recent years, seizure prediction has grown in popularity as a result of the advancements in machine learning and deep learning. Fei et al. used the improved Lyapunov exponent algorithm to better capture the subtle chaotic dynamics of epileptic signals in the fractional Fourier transform domain. Compared with the Traditional, Lyapunov exponent algorithm, the model has higher accuracy [4]. Raghu et al. showed that the successive decomposition index (SDI) increased significantly during seizures, Therefore, they proposed a successive decomposition index (SDI) feature that predicts seizures based on changes in SDI before onset [5]. Bandarabadi et al. used the feature selection of amplitude distribution. By calculating the amplitude distribution histograms (ADHs) of epileptic EEG sample features, ranking each feature, and then selecting features with the largest ADHs difference [6]. Wang and Lyu proposed a feature selection according to elimination and combined it with SVM to select the optimal feature set [7]. Yuan and Wei proposed a Bayesian linear discriminant analysis (BLDA) algorithm which is used as a classifier to determine the sample features. BLDA employs the regularization method in contrast to the conventional Fisher’s linear discriminant analysis in order to prevent the over-fitting issue [8]. Xu et al. presented an end-to-end one-dimensional convolutional neural network (CNN) architecture to directly input epileptic EEG signals into the CNN model [9]. Zhang et al. computed the Pearson correlation coefficient of the EEG signals to obtain the correlation matrix, which they then entered into the CNN model for classification [10]. A 3D CNN model was presented by Ozcan and Erturk to take use of the temporal and spatial correlation of EEG [11]. Abdelhameed and Bayoumi adopted a deep convolutional auto-encoder to identify the best spatial features from EEG signals and a BiLSTM for temporal information classification [12]. In order to extract the temporal and spatial characteristics of multi-channel EEG signals, a CNN-LSTM model for epileptic seizure prediction was put forth by Shahbazi and Aghajan [13]. Daoud and Bayoumi [30] use convolutional neural networks to extract significant spatial features from different scalp locations, use recurrent neural networks to predict seizures, and introduce a semi-supervised method based on transfer learning techniques to improve optimization problems.

To accurately forecast epileptic seizures, the goal of this study is to automatically extract the characteristics of epileptic EEG using deep learning. In order to make better use of the temporal and spatial characteristics of epileptic EEG signals. We propose a CBAM-3D CNN-LSTM model to predict seizures. First, we preprocess EEG signals using STFT. Secondly, the 3D CNN model was used to abstract the features of interictal stage and preictal stage from the preprocessed signals. Thirdly, Bi-LSTM is connected to 3D CNN for classification. Finally, CBAM is introduced into the model to give different attention to the channel and space of the data to extract the key information, so that the model can accurately extract the interictal and pre-ictal features, and improve the learning ability and robustness of the model. Our proposed approach achieved 97.95% accuracy, 98.40% sensitive, and 0.017 h^−1^ false alarm rate on 11 patients from the public CHB-MIT scalp EEG dataset.

This article is structured as follows: Section II describes the materials and methods, including data sets, data set preprocessing, 3D CNN, Bi-LSTM, CBAM, training and testing methods. Section III shows the experimental results and comparison with other experimental models. Section IV discusses the experimental results and models.

## II. Methodology

**FIGURE 3.**
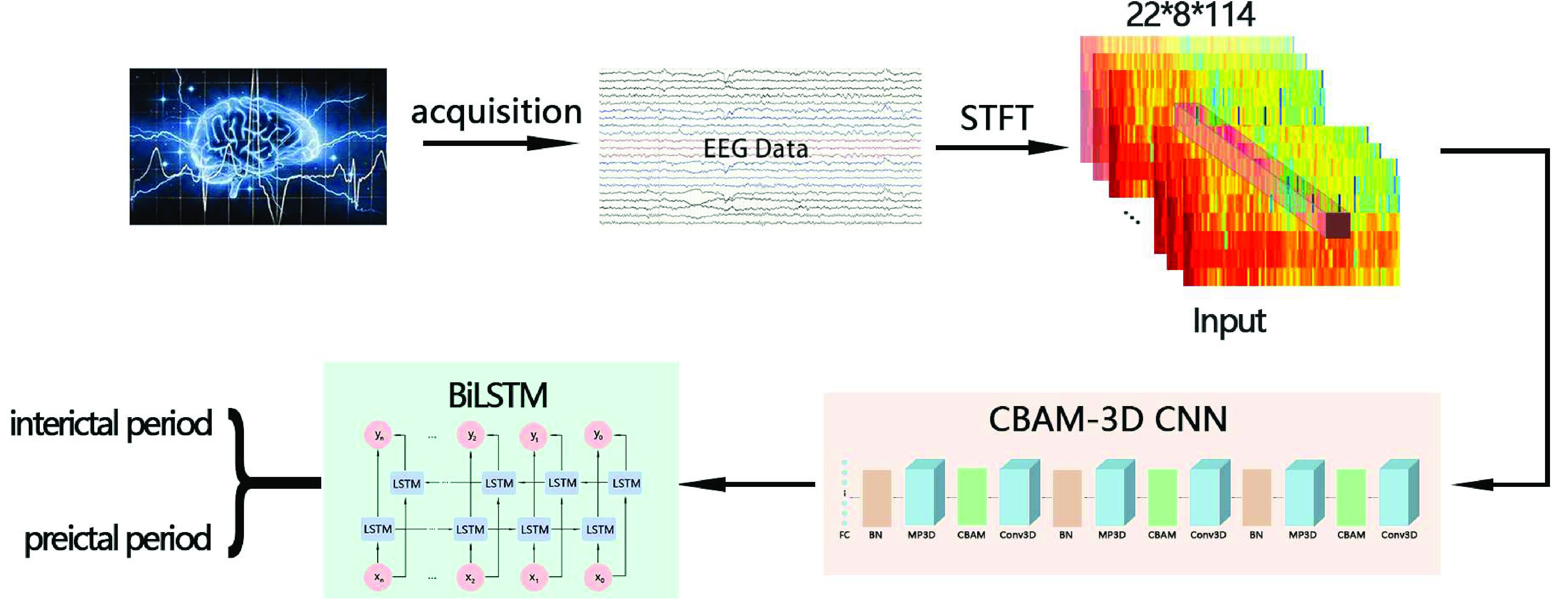
Schematic of epilepsy prediction model.

Epilepsy EEG signal is the result of time and space evolution, so its time and space has a certain correlation. Usually, the CNN model used in the epilepsy prediction method refers to the 2DCNN model, which has certain advantages in extracting spatial features, but ignores the temporal features of EEG signals. The LSTM model is more suitable for processing timing information. Both models cannot make good use of the temporal and spatial correlation of epileptic EEG signals. Therefore, inspired by deep learning in video processing [25], human behavior recognition [26] and sEMG noise recognition, figure 3 illustrates our suggested CBAM-3D CNN-BiLSTM seizure prediction model. Firstly, the collected data set is marked and preprocessed, and then the processed data set is extracted by 3D CNN model, and CBAM is introduced into the model to improve the learning ability and robustness of the model. Finally, BiLSTM utilized to classify the stages of interictal and preictal.

### A. Dataset

In this study, we make use of the epileptic EEG data set (CHB-MIT), which was co-created and recorded by scientists from MIT and Boston Children’s Hospital. The CHB-MIT dataset [14] is the most common public dataset for seizure detection and seizure prediction. It consists of 23 incidents and 844 hours of nonstop scalp EEG data from 22 pediatric patients. EEG data from 22 electrodes were collected at a sampling rate of 256 Hz using the bipolar montage technique of the international 10–20 system. The start and finish times of each seizure are clearly annotated in the dataset.

In our study, we used continuous EEG signals from 35 minutes to 5 minutes before a seizure as a pre-seizure. The 10 minutes after the end of seizures as post - seizure EEG. The interictal period is defined as the period between 4 hours after the end of the seizure and 4 hours before the start of the next seizure. In addition, to carry out intervention in therapy, we took the EEG signal 5 minutes before the attack as the intervention period and deleted it from the data. Due to the requirements of model training and testing, the number of seizures should not be less than 3 times and not more than 10 times

Therefore, we selected 11 patients, 55 seizures, and 235 hours of continuous EEG from the CHB-MIT dataset, as shown in Table 1.

**TABLE 1:** The Number of Seizures and the Duration of Interphase Data of 11 Patients in the CHB-MIT Dataset

Patient	Quantity of seizures	Iterictal hours/h
Pt 01	7	14.4
Pt 02	3	26.1
Pt 03	6	27.4
Pt 05	5	14.5
Pt 08	4	1.0
Pt 09	4	48.6
Pt 10	7	24.2
Pt 13	7	15.1
Pt 19	3	26.0
Pt 21	4	23.5
Pt 23	5	14.2
Total	55	235.0

### B. Preprocessing

**FIGURE 4.**
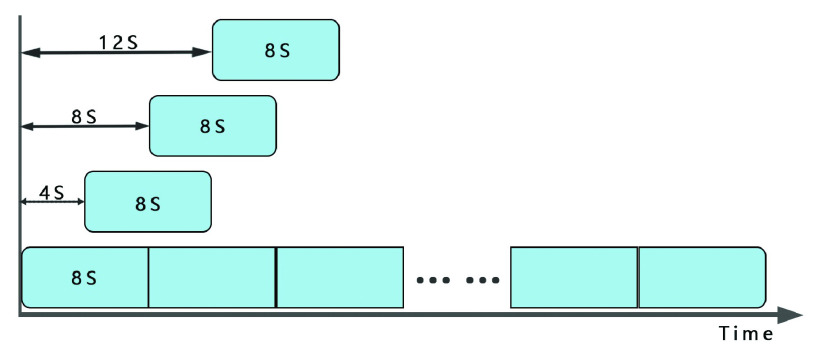
Overlapped sliding window sampling technique.

As a result of the vastly greater number of interictal data than preictal data, and the deep learning model is not suitable for dealing with a data imbalance problem. Therefore, we aim to balance the interictal and preictal datasets. We use overlapping sampling techniques to generate more training data sets, and separate EEG segments from continuous scalp EEG signals to select 8-second long overlapping activity windows. The sliding length is 4s, as shown in Figure 4. N fragments (N is the number of pre-ictal datasets) were randomly selected from the interictal data as the training set for the interictal period.

In the study, we needed to use the 3D CNN model to extract features from the dataset, so we needed to convert the EEG signal into a spectrogram. Fourier transform and wavelet transform are commonly used methods to convert EEG signals into spectrograms in epilepsy detection and prediction.

**FIGURE 5.**
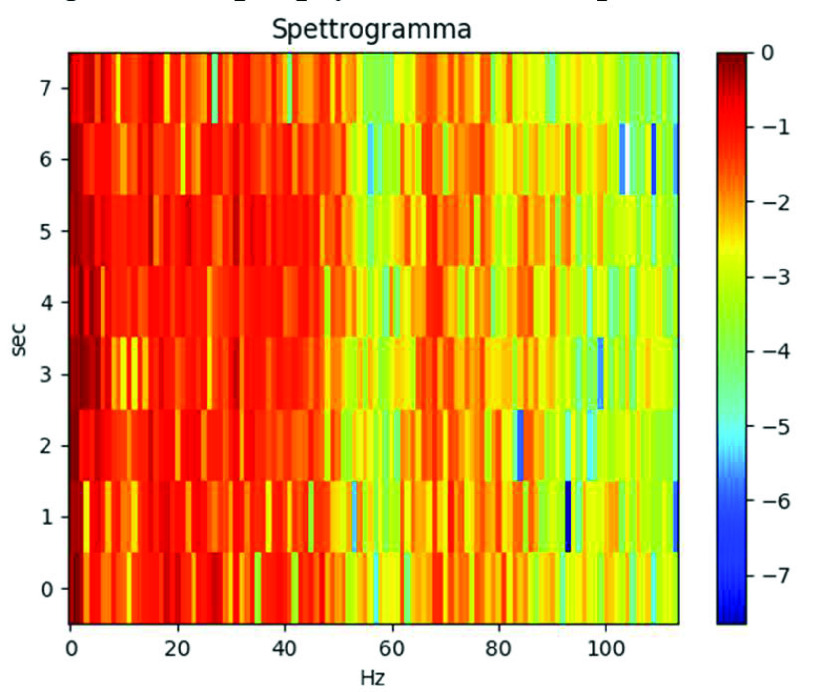
8 s EEG signal de-noising spectrum after short time fourier transform.

The majority of the EEG signals captured by the CHB-MIT dataset have 60 Hz power line noise interfering with them. Therefore, we use band-stop filter and high-pass filter to eliminate 57-63Hz and 117-123Hz frequency components to remove noise, and also remove 0Hz DC component. 8 s EEG Signal De-noising Spectrum after Short Time Fourier Transform, as shown in Figure 5.

### C. 3D CNN

A type of feedforward neural network with convolution calculation and a deep structure is a convolutional neural network [15]. It is one of the key deep learning methods and is widely used in image classification, speech recognition, machine vision, and other domains. CNN holds the capacity to represent learning, according to its hierarchical structure of the input information translation invariant classification, it is also referred to as ’ translation invariant artificial neural network ’ [16]. CNN is mainly composed of convolution layer, pooling layer and fully connected layer. The convolution layer is typically employed to extract features from the input data and output the feature map. Then, it is down-sampled through the pooling layer to reduce the dimension of the feature and reduce the computational complexity. Finally, the output data from the preceding layer is then applied to the fully connected layer to create a one-dimensional feature vector [29].

**FIGURE 6.**
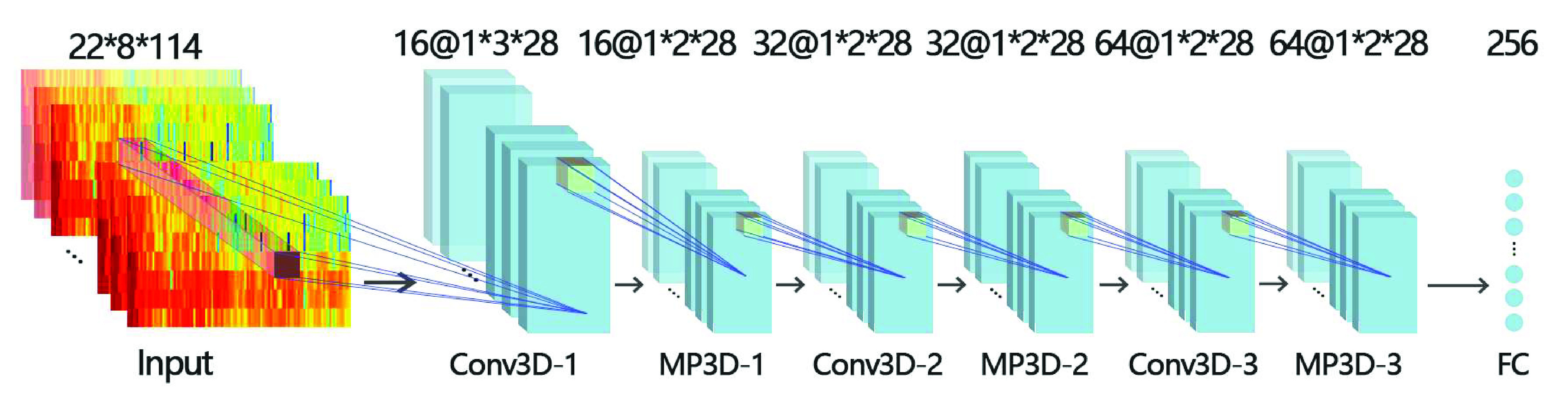
3D CNN model.

Since epileptic seizure is the result of its temporal and spatial evolution, in order to make better use of the temporal and spatial characteristics of epileptic EEG signals. We propose a 3D CNN model as a feature extractor. The model has three convolutional layers, three maximum pooling layers and a fully connected layer, as shown in Figure 6.

In the first convolution stack, the convolution kernel size is 22 * 3 * 3 and the pooling layer size is 1 * 2 * 2. In the other two convolution stacks, the size of convolution layer and pooling layer is 1 * 1 * 1. All convolutional layers use the RELU activation function, and use Batch Normalization to increase the model’s capacity for generalization, suppress the model overfitting, and improve the training speed of the model.

### D. Bi-LSTM

Recurrent convolutional neural network (RNN) is one kind of neural network with sequence data as input, recursion in the evolution direction of sequence and all recurrent units connected in chain. RNN is suitable for time series data prediction. It can process the time series of data by processing the previous sequence. However, RNN has the disadvantages of gradient explosion and disappearance and information deformation in the process of back propagation training over time.

**FIGURE 7.**
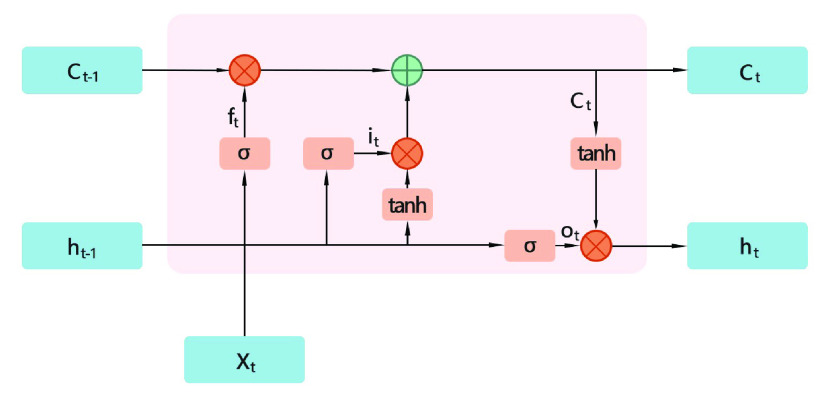
LSTM unit structure.

LSTM can scale the gradient value during training and solve the problem of gradient disappearance through time back propagation. The LSTM consists of three gates, the input, the forgetting, and the output gate. The three gates create a self-circulation of the internal state of the LSTM unit, as shown in Figure 7.

The update expression of the LSTM unit is as follows [17]:

\begin{align*} h^{(t)}&=g_{0}^{\left ({t }\right)}f_{h}\left ({s^{\left ({t }\right)} }\right) \tag{1}\\ s^{(t)} &=g_{f}^{(t)}s^{(t-1)}+g_{i}^{(t)}f_{s}(wh^{(t-1)}uX^{(t)}+b) \tag{2}\\ g_{i}^{(t)}&=sigmoid(w_{i}h^{(t-1)}u_{i}X^{(t)}+b_{i} \tag{3}\\ g_{f}^{(t)}&=sigmoid(w_{f}h^{(t-1)}u_{f}X^{(t)}+b_{f} \tag{4}\\ g_{o}^{(t)}&=sigmoid(w_{o}h^{(t-1)}u_{o}X^{(t)}+b_{o} \tag{5}\end{align*}

In the formula, Footmarks $i$, $f$, $o$ represent input gate, forgetting gate and output gate. $f_{h},f_{s}$ are the excitation functions of system state and internal state, usually a hyperbolic tangent function. G is a gated control updated over time.

**FIGURE 8.**
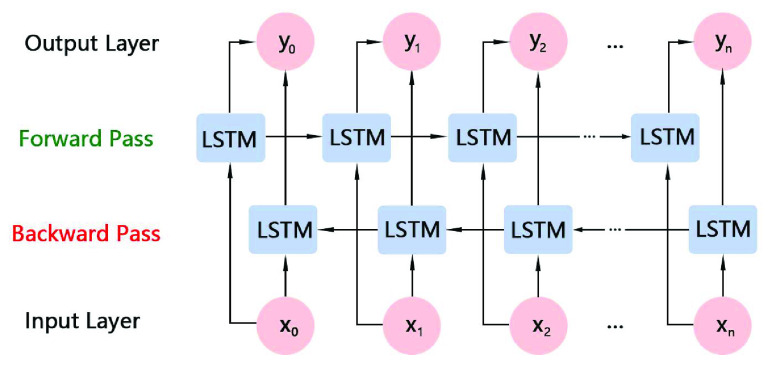
Bidirectional long short-term memory.

This study employs the Bi-LSTM [18] classifier, which processes the time series in two opposing orientations while substituting two blocks for each LSTM block, as illustrated in Figure 8. The feature vector generated from the 3D CNN model is input into the forward transfer block of Bi-LSTM from the beginning to the end of its first instance, and then the same fragment is processed in the opposite order. Each time step’s combined output from its two blocks is what is known as the network output for that step. Compared to LSTM, Bi-LSTM can handle both previous and future contexts, thus enhancing prediction results. In the Bi-LSTM classification process, to prevent overfitting, we employ the Dropout regularization technique. Dropout is applied with a 50% factor to the input and loop states. As the cost function, the cross entropy loss function is employed and Adma is selected as the optimizer for optimization.

### E. CBAM

The attention mechanism originates from the study of human vision. Later, it is gradually applied to the field of deep learning algorithms. The attention mechanism adjusts the network parameters by generating and assigning weights, and its role is to allocate computing resources to relatively more important tasks [27].

**FIGURE 9.**
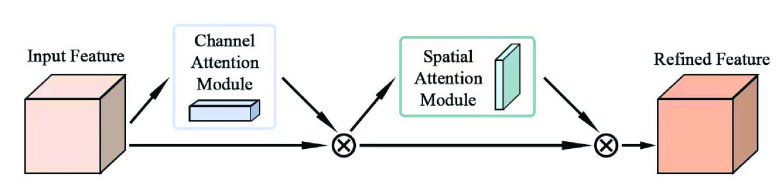
Convolutional block attention module.

In this paper, Convolutional Block Attention Module (CBAM) is used to optimize the seizure prediction model. CBAM is a multi-attention mechanism proposed by Woo et al. [28] in 2018. It connects the channel attention module and the spatial attention module in series. Compared with the attention mechanism that only focuses on a single aspect, CBAM focuses on both channel and spatial attention, which can achieve better results. The structure of CBAM is shown in Fig 9.

**FIGURE 10.**
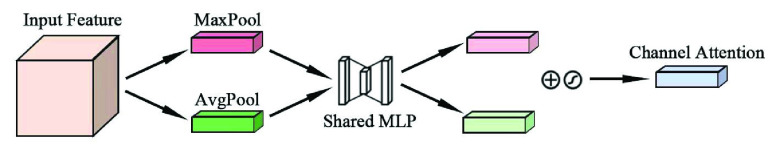
Channel attention module.

The structure of the channel attention module is shown in Figure 10. Firstly, the input features are subjected to global maximum pooling and global average pooling operations, and then jointly input into the multi-layer perceptron network. Next, the output features are sequentially added and activated by the sigmoid function to obtain the channel attention feature weight, and then multiplied with the input features to generate the features required by the spatial attention module.

**FIGURE 11.**
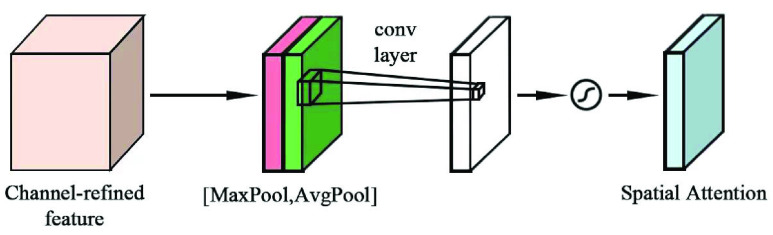
Channel attention module.

The structure of the spatial attention module is shown in Figure 11. The input feature map is subjected to channel-based global maximum pooling and global average pooling operations, and the obtained feature map is subjected to channel splicing. After convolution operation and sigmoid function activation operation, the spatial attention feature weight is generated, and it is multiplied with the input feature map to generate the final feature.

### F. Training and Testing Methods

In order to simulate the real situation, and to avoid over-fitting and model robustness, we evaluated our suggested model using the leave-one-out cross-validation approach (LOOCV) [19] for each patient. In other words, we select one seizure from a patient’s total of N seizures as the test set, and the model is trained using the remaining N-1 seizures. As a result, this procedure will be carried out N times. In this study, 25% of the data from the pre-ictal and interictal samples were randomly chosen as the test set and 75% were chosen as the training set.In the training process, we select a larger number of iterations to increase the accuracy of training, but it is easy to cause over-fitting during the training process. Therefore, we use the early-stop method to solve this problem, stopping training immediately when the validation set accuracy reaches 99 % or the validation set loss function begins to increase.

## III. Results

**FIGURE 12.**
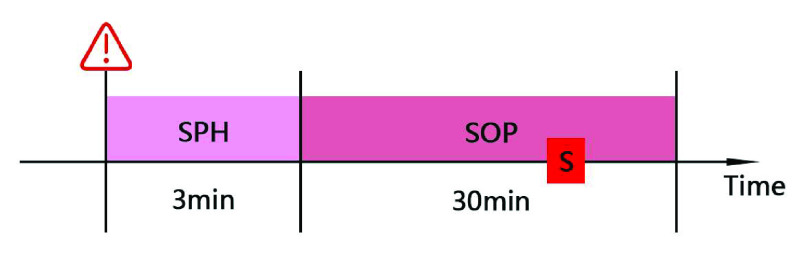
SPH and SOP.

To assess how well seizure prediction algorithms function, two important time parameters were defined during the study: seizure occurrence period (SOP) and seizure prediction horizon (SPH), as shown in Figure 12. SPH refers to the period between the time point when the predictive alarm is issued and the time point when the SOP starts. At the same time, appropriate means can be used to deal with seizures at this stage. Therefore, SPH is also called clinical intervention [20]. SOP is the period when seizures occur.

For a correct prediction of epilepsy, it should be within the SPH range after the alarm is issued, no seizures occur, while seizures occur within the SOP range, and the specific time point of the attack can be different. All other things are wrong. Therefore, to assess the effectiveness of seizure prediction models, different ranges of SPH and SOP need to be defined. For example, the smaller the SOP, the more accurate the prediction of the upcoming epileptic seizure time point. The ideal situation is that the SOP is reduced to a time point, which means that the epileptic seizure is accurately generated at this time point. However, it is particularly difficult to design such a prediction model. There is no perfect prediction model that can accurately predict a certain time point of epileptic seizures in patients. Therefore, SOP is not the smaller the better. As the SOP range decreases, the number of false predictions increases. In addition, the researchers believe that although the scope of the SPH definition of the larger the number of false positives, but the SPH range will increase the patient ’s anxiety, to bring a heavy psychological burden on patients [21]. Therefore, SOP was set at 30 minutes and SPH to 3 minutes for this study.

In this study, we also used three parameters: accuracy, sensitivity and false prediction rate (FPR) as the evaluation indexes of epileptic seizure prediction model. Among them, sensitivity and FPR are two key evaluation indicators that researchers are most interested. Sensitivity is the prediction model’s capacity to recognize the pre-epileptic phase of the EEG with accuracy., and FPR is a measure of how many incorrect predictions the model makes each hour [6]. Its mathematical expression is as follows.

\begin{align*} \mathrm {Accuracy}&=\frac {TP+TN}{TP+TN+FP+FN} \tag{6}\\ \mathrm {Sensitivity}&=\frac {TP}{TP+FN} \tag{7}\\ \mathrm {FPR}&=\frac {FP}{Time} \tag{8}\end{align*}

Among them, TP was true positive, FP was false positive, TN was true negative, and FN was false negative.

Table 2 shows the accuracy, sensitivity and FPR prediction results of our model for 11 patients. We can see that the average performance of the proposed model reaches 97.95 % accuracy, 98.40 % sensitivity and 0.017 h^−1^ FPR on the CHB-MIT dataset. Among all patients, Pt01 and Pt08 achieved very good results, reaching more than 99 % accuracy, 100 % zero sensitivity and 0 false prediction rate, and achieved good results in other patients.

**TABLE 2:** Results Obtained Using Our Model for Each Case in the CHB-MIT Dataset

Patient id	ACC	SEN	FPR/h^−1^
Pt 01	99.16%	100%	0.00
Pt 02	96.44%	92.68%	0.06
Pt 03	98.03%	99.55%	0.02
Pt 05	97.59%	99.73%	0.03
Pt 08	99.04%	100%	0.00
Pt 09	98.45%	99.66%	0.03
Pt 10	97.28%	100%	0.01
Pt 13	97.13%	98.82%	0.03
Pt 19	98.23%	97.10%	0.02
Pt 21	97.14%	96.35%	0.02
Pt 23	99.06%	98.53%	0.00
Average	97.95%	98.40%	0.017

Table 3 shows the comparison results of 3DCNN model and time prediction model. We can see that the model combining 3DCNN with BiLSTM has better accuracy, sensitivity and FPR than the model combining 3DCNN with BiGRU. Therefore, we use the 3DCNN-BiLSTM model to predict seizures. At the same time, we introduce CBAM into 3DCNN-BiLSTM. It can be seen from Table 3 that the CBAM-3DCNN-BiLSTM model achieves better results than the other two models.

**TABLE 3:** The Comparison Results of 3DCNN Model and Time Prediction Model are Compared

Model	ACC	SEN	FPR/h^−1^
3DCNN-BiGRU	93.21%	90.11%	0.09
3DCNN-BiLSTM	94.86%	91.87%	0.06
This Work	97.95%	98.40%	0.017

The performance comparison between our model and the formerly suggested deep learning-based seizure prediction technique is shown in Table 4. The CHB-MIT dataset is used to assess all methodologies. The table shows that, in comparison to other models, our model achieves high accuracy, high sensitivity, and a low false prediction rate. Therefore, our proposed CBAM-3DCNN-BiLSTM model is significantly superior to other CNN-based methods.

**TABLE 4:** Comparison With Other Model Experiments

Model	ACC	SEN	FPR/h^−1^
CNN [21]	81.2%	75%	0.16
CNN [23]	–	87.8%	0.14
3DCNN [11]	–	85.7%	0.096
3DCNN [23]	80.5%	85.8%	–
This Work	97.95%	98.40%	0.017

## IV. Conclusion

In this study, a CBAM-3DCNN-BiLSTM model for seizure prediction was suggested. EEG signals are transformed into three-dimensional feature vectors using the STFT algorithm. Time, frequency, and channel data are used to extract features using 3DCNN. CBAM is introduced into the model to filter important node information, avoid feature redundancy, and improve model learning ability and robustness, and BiLSTM is used to classify the extracted features. This method achieves 97.95 % accuracy, 98.40 % sensitivity and 0.017 h^−1^ FPR. Compared with the previous work, the experimental results show that the proposed method has high accuracy and sensitivity, and low error prediction rate. The epileptic seizure prediction method in this study is superior to other methods. Due to the patient-specific nature of epileptic EEG data, we need to test more subjects in different age groups, different clinical conditions, and different disease characteristics in future work to ensure that the method can be promoted. At the same time, through the existing technology to continuously improve the accuracy of its prediction, reduce the potential risk of epilepsy patients, and protect the life and health of epilepsy patients.
